# Electro-Quasistatic Analysis of an Electrostatic Induction Micromotor Using the Cell Method

**DOI:** 10.3390/s101009102

**Published:** 2010-10-11

**Authors:** José Miguel Monzón-Verona, Francisco Jorge Santana-Martín, Santiago García–Alonso, Juan Antonio Montiel-Nelson

**Affiliations:** 1 University of Las Palmas de Gran Canaria, Department of Electrical Engineering, Institute for Applied Microelectronics, Campus Univ. de Tafira s/n, 35017 Las Palmas de Gran Canaria, Spain; E-Mails: jmonzon@die.ulpgc.es (J.M.M.-V.); fsantana@die.ulpgc.es (F.J.S.-M.); 2 University of Las Palmas de Gran Canaria, Department of Electronic Engineering and Automatic, Institute for Applied Microelectronics, Campus Univ. de Tafira s/n, 35017 Las Palmas de Gran Canaria, Spain; E-Mail: sgarcia@diea.ulpgc.es (S.G.-A.)

**Keywords:** micromotor, electrostatic, induction, cell method, direct finite formulation

## Abstract

An electro-quasistatic analysis of an induction micromotor has been realized by using the Cell Method. We employed the direct Finite Formulation (FF) of the electromagnetic laws, hence, avoiding a further discretization. The Cell Method (CM) is used for solving the field equations at the entire domain (2D space) of the micromotor. We have reformulated the field laws in a direct FF and analyzed physical quantities to make explicit the relationship between magnitudes and laws. We applied a primal-dual barycentric discretization of the 2D space. The electric potential has been calculated on each node of the primal mesh using CM. For verification purpose, an analytical electric potential equation is introduced as reference. In frequency domain, results demonstrate the error in calculating potential quantity is neglected (<3‰). In time domain, the potential value in transient state tends to the steady state value.

## 1. Introduction

Electromagnetic laws were formulated *ab initio* using global quantities, such as charge, current, electric and magnetic flux, electromotive and magnetomotive force. Kirchoff’s network equations were also stated using global quantities, potential and current.

After Maxwell’s publication, electromagnetic laws have been commonly written using differential equations. Because differential formulation is restricted to homogeneous regions—material homogeneity—heterogeneous domains are broken in homogeneous subdomains plus jump conditions. The discrete formulation of differential equations requires a discretization method, such as finite difference, finite element, boundary element, among others.

**Figure 1.**
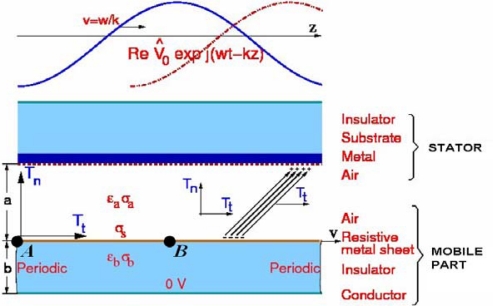
Linear electrical induction micromachine.

As an alternative, a direct Finite Formulation (FF) of the electromagnetic laws based on global variables accepts material discontinuities, as is the case of the micromotor interface region, which is the surface of the resistive metal sheet of the mobile part of the micromotor in contact with the air (see Figure 1). In a direct FF [1–3], an algebraic system of equations is directly written, avoiding the discretization process. The corresponding numerical method is known as the Cell Method (CM) [4–6]. The present paper applies this method to the simulation and analysis of an electrostatic induction micromotor.

The main benefit of CM is the remarkable simplification of its theoretical formulation, and therefore, the obtained equation system. The CM algebraic equation system is equivalent to the obtained in FEM using affine approximation of the electric potential inside of the triangle mesh. CM simplification is because physical laws of the electrostatic induction micromotor are expressed directly by a set of algebraic equations. However, in FEM, the algebraic equations are obtained after a discretization process using differential equations. Thus, CM requires two steps less than FEM to obtain the same algebraic system of equations.

The fundamental principle of CM is the use of finite or global measurable quantities. In the micromotor analysis, we use the voltage along a line instead of the electric field in a point. Therefore, we don’t use those quantities that are defined through a mathematical limit process as standard operations of gradient, curl and divergence. Note that a mathematical limit process involves operational difficulties in some conditions—such as discontinuities in the electrical field in the interface, due to the superficial conductivity. They are not adequate for numerical processing. Because of this, FEM involves two additional steps: first, Green’s theorem is applied; and second, the first order interpolation function of Whitney elements is used. The last step introduces a tangential continuity of the field magnitudes in the edge of the elements and, however, allows discontinuity in the normal component. The constitutive equations in CM formulation have a deep geometric interpretation based in the geometry of primal and dual meshes. This interpretation facilitates the incorporation of two types of physical properties, volumetric and superficial with electrical conductivity.

Nowadays, the design and implementation of a micromotor using MEMS technology is a great challenge [7–9]. For this purpose, we have developed some tools based on FF to simulate the electromagnetic fields of an electrostatic induction micromotor. In [10], we introduce the analytical equations for an electrostatic induction micromotor. References [11–15] provide state-of-the-art contributions in discrete electromagnetism and electrostatic formulation. In [12], its authors apply CM for computing the capacitance of a transmission line in presence of non homogeneous media. Reference [13] deals with a general application of CM to solve both isotropic and anisotropic electrostatic problem. In [12,13], the dielectric is characterized by a constitutive permittivity matrix—volumetric property—; the electric conductivity is neglected at the whole domain.

In this work, for an electrostatic micromotor, the superficial conductivity at the interface of the mobile part plays a key role. In addition, we consider a volumetric conductivity at the mobile part. Figure 1 illustrates both superficial and volumetric conductivity, σ_S_ and σ_b_, respectively. As a consequence, time dependent terms are considered in our finite formulation problem, and therefore, we carry out both frequency and transitory analysis.

The analyzed micromotor is a simple linear electrostatic induction micromachine constituted by two parallel plates—mobile part and stator—isolated by a dielectric [8]. The distance between plates is 6 μm. Figure 1 summarizes the operation mode of the micromachine. Table 1 shows the nomenclature and Table 2 presents the physical and geometrical parameters of the micromachine. Our work is focused in the linear micromachine due to the great simplicity of analytical equations. The linear micromachine is the unfolding of a rotating electric micromachine. Consequently, the conclusions obtained for the linear micromachine are directly generalized to the rotating one [16].

The paper is organized as follows. Section 2 contains the reformulation of the field laws in a direct FF for the micromotor. Initially, we introduce global variables by analyzing physical quantities in order to make explicit the maximum of information. Both topological and constitutive equations are explained in detail. Then, we present the final global equation of the electrostatic induction micromotor. In Section 3, we provide an analytical equation of the electric potential—global variable—at the interface of the micromotor. For verification purpose, electric potential values are calculated by solving field equations with CM. Both frequency and time domain comparisons are introduced. Finally, Section 4 provides conclusions of the work.

## 2. Finite Formulation for the Micromotor

The reformulation of field laws in a direct FF begins with an analysis of physical quantities. Physical measurements deal with global variables against field variables. In differential formulation, field variables are utilized because the notion of derivative refers to a point function. Contrariwise, global variables refer to a system, at a space or time element—global variables concern to oriented geometrical elements like points, lines, surfaces, volumes and time elements like instant and interval.

According to FF, global variables are also classified into configuration, source, and energy variables [1]. The configuration variables describe the configuration of the field without the intervention of the material parameters. The source variables describe the source of the field without involving the material parameters. The energy variables are the product between a configuration and source variable.

**Figure 2.**
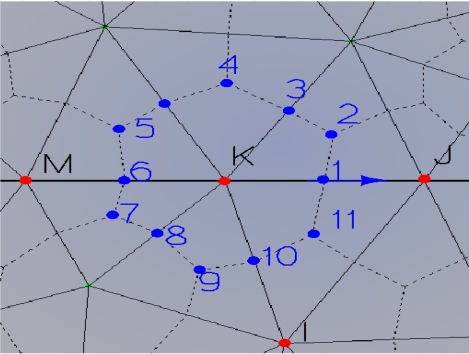
Dual barycentric subdivision.

CM requires the use of a pair of oriented cell complexes, one dual to each other, endowed with inner orientation (see I,J,K cell in Figure 2) and outer orientation (see 1,2,3,...,11 cell in Figure 2). Figure 2 illustrates the corresponding dual cell complexes. They were obtained from the barycentric subdivision [11].

According to electromagnetism FF, the first principle [3] says that the configuration variables are naturally associated with space and time elements of a primal cell complex endowed with inner orientation, while the source variables are associated with space and time elements of a dual cell complex endowed with outer orientation. The second principle [3] says that in every physical theory there are physical laws that link global variables referred to an oriented space-time element with others referred to its oriented boundary.

### 2.1. Topological Equation of the Micromotor in Discrete Form

The field equation of the micromotor is enforced, on the cell complexes, in exact discrete form by using incidence matrices *G*, *C* and *D*. They are denoted as edges-nodes, faces-edges, and volumes-faces, respectively, for the oriented primal cell complex. Let matrices *G͂*, *C͂* and *D͂* denote the node-edges, edges-faces, and faces-volumes, respectively, for the oriented dual cell complex. These matrices are viewed as discrete counterparts of the differential operator gradient, curl, and divergence [5,12,14].

The following equations represent the counterparts of the differential Maxwell′s laws.

Gauss law:

(1) $$\tilde{D}\tilde{\psi }=\tilde{Q}$$

where *ψ͂* is the electric flux vector associated to the dual faces and *Q͂* the electric charge vector associated to the dual volumes.

Faraday law (for quasi–electrostatic conditions):

(2) CU=0

(3) U=−GV

where *V* is the electric potential associated to the primal nodes and *U* the voltage vector associated to the primal edges.

Charge conservation law:

(4) $$\tilde{D}\tilde{I}+\tilde{D}\frac{d\tilde{\psi }}{\mathrm{dt}}=0$$

where *Ĩ* is the electric current vector associated to the dual faces.

The duality between the oriented primal and dual space cell complexes leads, in general, to the following relationships [14,17]:

(5) $$\tilde{D}=-G^{T}$$

(6) $$\tilde{C}=C^{T}$$

(7) $$\tilde{G}=D^{T}$$

### 2.2. Constitutive Equation of the Micromotor in Discrete Form

While field equations in direct FF describe the physical laws exactly, the constitutive equations describe the physical laws approximately. For the micromotor the integral potential and flux state variables, which are allocated on two different cell complexes, are related to each other by the constitutive material equations. These equations are matrix equations. They contain the average information of the material and grid dimension [6,11,12,18–20].

Since Equations (1)–(4) only contain topological information, the discretization error comes from the discrete constitutive material equations.

Volumetric properties—volumetric conductivity and permittivity—and superficial properties—superficial conductivity—are considered constitutive equations of the micromotor. Therefore, two classes of cells for the discrete constitutive material equations rise. For a bidimensional form, the volume cell and face cell are transformed in face cell and edge cell, respectively.

**Figure 3.**
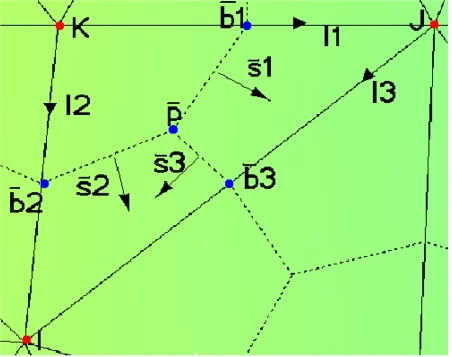
Simple primal-dual cell for assemble process.

The constitutive equations for a simple primal–dual cell (see Figure 3), are as follows:

(8) I˜e=MUσee

(9) ψ˜e=MUɛee

The expressions for the face element are:

(10) $${\tilde{\psi }}^{e}={\left({\tilde{\psi }}_{1}\;\;{\tilde{\psi }}_{2}\;\;{\tilde{\psi }}_{3}\right)}^{T}$$

(11) $${\tilde{I}}^{e}={\left({\tilde{I}}_{1}\;\;{\tilde{I}}_{2}\;\;{\tilde{I}}_{3}\right)}^{T}$$

and for the edge element:

(12) $$U^{e}={\left(U_{1}\;\;U_{2}\;\;U_{3}\right)}^{T}$$

$M_{\sigma_{V}}^{e}$ and $M_{{ɛ}_{V}}^{e}$ are the volumetric conductivity and the permittivity matrices, respectively:

(13) $$M_{\sigma_{V}}^{e}=1/3{\tilde{S}}^{e}\sigma^{e}\left(A^{e}+B^{e}+C^{e}\right)$$

(14) $$M_{{ɛ}_{V}}^{e}=1/3{\tilde{S}}^{e}{ɛ}^{e}\left(A^{e}+B^{e}+C^{e}\right)$$

*A^e^*, *B^e^* and *C^e^* depend on geometry of the primal cell and are expressed as follows:

(15) $$A^{e}=\frac{1}{\Delta_{1}}\left[\begin{array}{ccc} 0 & I_{3y} & -I_{2y}\\ 0 & -I_{3x} & I_{2x} \end{array}\right]$$

(16) $$B^{e}=\frac{1}{\Delta_{2}}\left[\begin{array}{ccc} I_{3y} & 0 & -I_{1y}\\ -I_{3x} & 0 & I_{1x} \end{array}\right]$$

(17) $$C^{e}=\frac{1}{\Delta_{3}}\left[\begin{array}{ccc} I_{2y} & -I_{1y} & 0\\ -I_{2x} & I_{1x} & 0 \end{array}\right]$$

Δ_1_, Δ_2_, and Δ_3_ expressions are:

(18) $$\Delta_{1}=I_{2x}I_{3y}-I_{2y}I_{3x}$$

(19) $$\Delta_{2}=I_{1x}I_{3y}-I_{1y}I_{3x}$$

(20) $$\Delta_{3}=I_{1x}I_{2y}-I_{1y}I_{2x}$$

(*I*_1x_, *I*_1y_), (*I*_2x_, *I*_2y_) and (*I*_3x_, *I*_3y_), are vectors associated to the primal edges:

(21) $$\left(I_{1x},I_{1y}\right)=\left(x_{j}-x_{k},y_{j}-y_{k}\right)$$

(22) $$\left(I_{2x},I_{2y}\right)=\left(x_{i}-x_{k},y_{i}-y_{k}\right)$$

(23) $$\left(I_{3x},I_{3y}\right)=\left(x_{i}-x_{j},y_{i}-y_{j}\right)$$

where (*x_i_*, *y_i_*) are the coordinates associated to the nodes of the triangle of reference (see Figure 3). *S͂^e^* is expressed as follows:

(24) $${\tilde{S}}^{e}=\left(\begin{array}{ll} {\tilde{S}}_{1x} & {\tilde{S}}_{1y}\\ {\tilde{S}}_{2x} & {\tilde{S}}_{2y}\\ {\tilde{S}}_{3x} & {\tilde{S}}_{3y} \end{array}\right)=\left(\begin{array}{l} {\tilde{S}}_{1}\\ {\tilde{S}}_{2}\\ {\tilde{S}}_{3} \end{array}\right)$$

The permittivity and conductivity tensors are:

(25) $${ɛ}^{e}=\left(\begin{array}{ll} {ɛ}_{11} & 0\\ 0 & {ɛ}_{22} \end{array}\right)$$

(26) $$\sigma^{e}=\left(\begin{array}{ll} \sigma_{11} & 0\\ 0 & \sigma_{22} \end{array}\right)$$

where *U*_1_, *U*_2_ and *U*_3_ are the voltage associated to the edges *I*_1_, *I*_2_ and *I*_3_ (see Figure 3), respectively; *ψ͂*_1_, *ψ͂*_2_, *ψ͂*_3_ and *Ĩ*_1_, *Ĩ*_2_, *Ĩ*_3_ are the electric flow and the electric intensity associated to the surfaces *S͂*_1_, *S͂*_2_ and *S͂*_3_, respectively, of the simple dual cell (see Figure 3).

$M_{\sigma_{s}}^{e}$ is the superficial conductivity matrix, and it is expressed as:

(27) $$M_{\sigma_{S}}^{e}=\frac{t^{e}\sigma_{S}^{e}}{l^{e}}$$

where $\sigma_{S}^{e}$ is the superficial conductivity and *l^e^* is the length of an element (*i*, *j*).

The components of the vectors associated to the dual surfaces *S͂*_1_, *S͂*_2_ and *S͂*_3_ (see Figure 3), are:

(28) $${\tilde{S}}_{1}=t\left(-P_{b1y},-P_{b1x}\right)$$

(29) $${\tilde{S}}_{2}=t\left(-P_{b2y},-P_{b2x}\right)$$

(30) $${\tilde{S}}_{3}=t\left(P_{b3y},-P_{b3x}\right)$$

where *t* stands for the thickness of the model, (*P_b_*_1_*_x_*, *P_b_*_1_*_y_*), (*P_b_*_2_*_x_*, *P_b_*_2_*_y_*) and (*P_b_*_3_*_x_*, *P_b_*_3_*_y_*) are vectors at the barycentric of the reference triangle:

(31) $$\left(P_{b1x},P_{b1y}\right)=\left(b_{1x}-{\tilde{P}}_{x},b_{1y}-{\tilde{P}}_{y}\right)$$

(32) $$\left(P_{b2x},P_{b2y}\right)=\left(b_{2x}-{\tilde{P}}_{x},b_{2y}-{\tilde{P}}_{y}\right)$$

(33) $$\left(P_{b3x},P_{b3y}\right)=\left(b_{3x}-{\tilde{P}}_{x},b_{3y}-{\tilde{P}}_{y}\right)$$

The centers (*b͂*_1_, *b͂*_2_ *b͂*_3_) of the edges 1, 2, and 3, respectively, are the coordinates:

(34) $$\left(b_{1x},b_{1y}\right)=\left(\frac{x_{k}+x_{j}}{2},\frac{y_{k}+y_{j}}{2}\right)$$

(35) $$\left(b_{2x},b_{2y}\right)=\left(\frac{x_{i}+x_{k}}{2},\frac{y_{i}+y_{k}}{2}\right)$$

(36) $$\left(b_{3x},b_{3y}\right)=\left(\frac{x_{i}+x_{j}}{2},\frac{y_{i}+y_{j}}{2}\right)$$

Point *P͂* is defined as:

(37) $$\left({\tilde{P}}_{x},{\tilde{P}}_{y}\right)=\left(\frac{x_{i}+x_{j}+x_{k}}{3},\frac{y_{i}+y_{j}+y_{k}}{3}\right)$$

### 2.3. Final Global Equation of the Micromotor

We obtain the local fundamental matrix by substituting in (4) the local constitutive Equations (8) and (9) and Gauss law (1), were *U* is expressed by means of (2) and (3):

(38) $$G^{\mathrm{eT}}M_{\sigma }^{e}G^{e}V^{e}+G^{\mathrm{eT}}M_{ɛ}^{e}G^{e}\frac{dV^{e}}{\mathrm{dt}}=0$$

where *G*^e^ is the incidence matrix of one element and is expressed as follows:

(39) $$G^{e}=\begin{array}{cc} & \begin{array}{lll} i & j & k \end{array}\\ \begin{array}{l} I_{1}\\ I_{2}\\ I_{3} \end{array} & \left(\begin{array}{lll} 0 & 1 & -1\\ 1 & 0 & -1\\ 1 & -1 & 0 \end{array}\right) \end{array}$$

and:

(40) $$V^{e}={\left(\begin{array}{ccc} V_{i} & V_{j} & V_{k} \end{array}\right)}^{T}$$

For computational purpose, processing cells one by one is convenient. To obtain the global fundamental matrix all the local fundamental matrices on the reference cell are assembled (see Figure 3).

For a bidimensional formulation, in case of triangular elements under the hypothesis of uniform field and using a dual mesh with barycentric subdivision, the resulting matrix for one element is symmetric. Moreover, this matrix is coincident with the element matrix obtained with finite elements with affine approximation of the electric potential within the triangle [4,12]. Therefore, the resulting system of equations is coincident. To solve Equation (38), we have applied the following boundary conditions, one travelling wave on top, 0 V at bottom; and periodic boundary conditions on the left and right side (see Figure 1).

## 3. Results

### 3.1. Discrete Field in Frequency Domain

First, Equation (38) is transformed to the frequency domain. Then, the operator *jω* substitutes to the operator *∂/∂t*. In this way, Equation (38) is expressed in the following form:

(41) $$G^{T}M_{\sigma }\mathrm{GV}+\mathrm{jw}G^{T}M_{ɛ}\mathrm{GV}=0$$

Next equation represents the analytical electric potential at the interface of the micromotor [10]. This is the reference equation for the FF verification.

(42) $$\Phi^{b}=\frac{V_{0}}{\text{sinh}(ka)}\frac{\frac{\sigma_{a}}{\sigma_{\mathrm{eff}}}+\frac{{ɛ}_{a}}{{ɛ}_{\mathrm{eff}}}\omega Sj}{\left(1+\frac{{ɛ}_{\mathrm{eff}}}{\sigma_{\mathrm{eff}}}\omega Sj\right)}$$

where:

(43) $$\sigma_{\mathrm{eff}}=\sigma_{a}\text{coth}(\mathrm{ka})+\text{coth}(\mathrm{kb})\sigma_{b}+\sigma_{S}k$$

(44) $${ɛ}_{\mathrm{eff}}={ɛ}_{a}k\text{coth}(\mathrm{ka})+{ɛ}_{b}k\text{coth}(\mathrm{kb})$$

**Figure 4.**
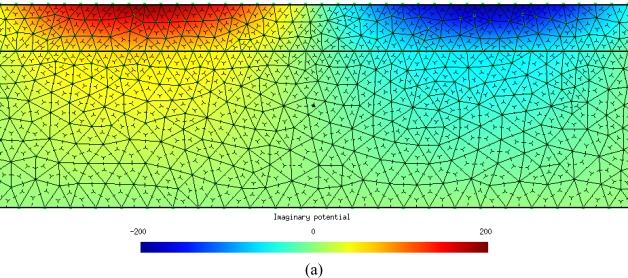

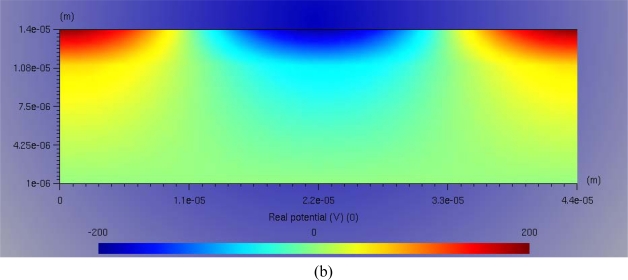
**(a).** Graphical representation of imaginary potential for a superficial conductivity of 1/(1800·10^6^) 1/Ω. **(b).** Graphical representation of real potential for a superficial conductivity of 1/(1800·10^6^) 1/Ω.

We have calculated the potential at the interface, applying CM and the analytical equations, for five different values of the conductivity. The mismatch between the results obtained using analytical equations and the CM are neglected, as can be seen in Table 3. Figure 4(a) and 4(b) show the CM results of the potential for a superficial conductivity of 1/(1800·10^6^) 1/Ω. Figure 4(a) and 4(b) represent the imaginary and real components of the scalar electric potential, respectively. Figure 4(a) also illustrates the primal and dual mesh.

**Figure 5.**
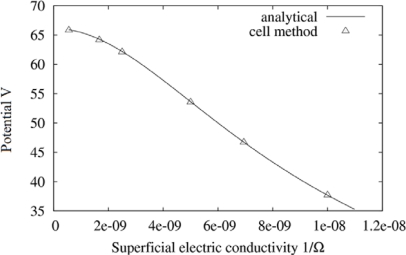
Graphical representation of maximal potential at the interface *versus* superficial electric conductivity.

Figure 5 represents the potential at the interface *versus* the conductivity. Typical maximum discrepancies are lower than 0.1%.

**Figure 6.**
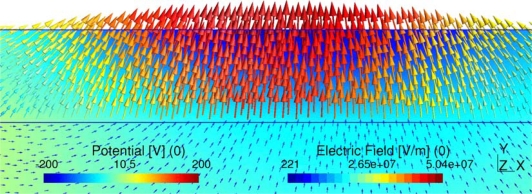
Electric field for a superficial conductivity of 1/(600·10^6^) 1/Ω.

We have also calculated the electric field at the interface. CM results and analytical solution results are illustrated in Table 4 and Figure 6 for a superficial conductivity of 1/(600·10^6^) 1/Ω. The error between the results obtained using analytical equations and the CM is neglected.

CM convergence has been guaranteed with the refining of the meshes of the micromotor as can be seen in Table 5. The interfacial electric potential has been obtained for a conductivity of 1/(1800·10^6^) 1/Ω.

### 3.1. Discrete Field in Time Domain

In order to perform numerical calculations in time domain it is necessary to discretize the time axis for Equation (38). The θ-method of integration [21–23] is widely used to calculate transient fields [24]. The accuracy of this method is usually lower or equal to second order methods. The θ-method is easily implemented and it has found wide application in transient analysis.

Equation (38) is an Ordinary Differential Equation (ODE) system [24] and it has the form:

(45) $$M\frac{\partial V}{\partial t}+\mathrm{NV}=f(t)$$

*M* and *N* are as follows:

(46) $$M=G^{T}M_{ɛ}G$$

(47) $$N=G^{T}M_{\sigma }G$$

where *f(t)* function represent the boundary conditions. In this method the time axis is divided into intervals *Δt*. The θ–method applied to solve Equation (45) is written as:

(48) $$M\frac{V_{n+1}-V_{n}}{\Delta t}+N(\theta V_{n+1}+(1-\theta )V_{n})=\theta f{\left(t\right)}_{n-1}+(1-\theta )f{\left(t\right)}_{n}$$

where index *n* and *n + 1* refer to quantities V at time t and *t + Δt*, respectively. Various choices of parameter *θ* lead to different classical methods. By *θ* = 1 the differential equation is solved by implicit Euler method, *θ* = 0 uses explicit Euler, *θ* = 0.5 is Crank–Nicholson method, *etc.* [25]. In this paper we considerate *θ* = 1.

**Figure 7.**
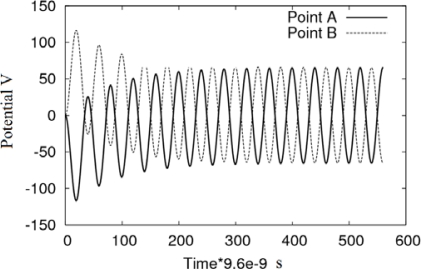
Transitory state of the potential at the interface in points *A* and *B.*

Figure 7 illustrates the transient state for the interface at *z* = 0 and *z* = *L*/2, *i.e.*, point *A* and *B* in Figure 1, respectively. At *t* = 0 the initial conditions for the electric potential is *V* = 0 for all the domain. The total time for the transient analysis is 14 cycles of the applied potential with a maximum value of 200 V. The time step used in θ-method is *T*/40 s, where *T* is the signal period of the applied potential (see Table 5). Note that the computed potential at the interface in transient state, tends to the value obtained in steady state, 65.9 V. Figure 7 shows that for the same instant, the potential magnitude at the points *A* and *B* is equal, but with opposite sign.

**Figure 8.**
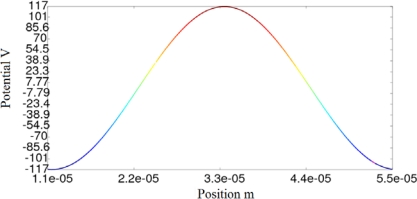
Transitory state of the micromotor: potential distribution at the interface for instant t_1_.

**Figure 9.**
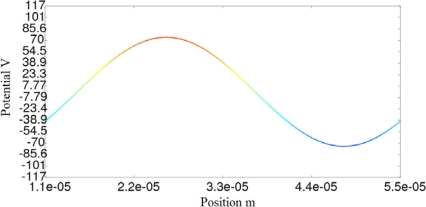
Transitory state of the micromotor: potential distribution at the interface for instant t_2_.

**Figure 10.**
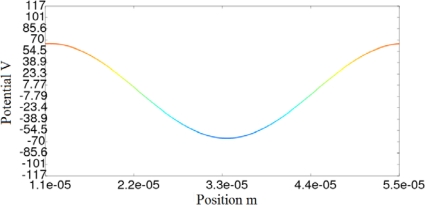
Transitory state of the micromotor: potential distribution at the interface for instant t_3_.

Figures 8–10 show the potential distribution at the interface for three time instants: t_1_ = 1.92·10^−7^ s, t_2_ = 4.81·10^−7^ s and t_3_ = 3.85·10^−6^ s, respectively. As the transient analysis evolves, the maximum value approaches to the value that will be reached in permanent state, as can be seen in Figure 10. We must also consider that in the first cycles of the signal, the traveling wave changes its aspect until it reaches the definitive sinusoidal shape that is represented in Figure 10.

## 4. Conclusions

As an alternative to the differential formulation of the electromagnetic laws, we have rewritten the field laws of an electrostatic induction micromotor in a direct finite formulation. For solving field equations in direct finite formulation, we applied the Cell Method (CM) and obtained a relationship between volumetric and superficial material properties at the interface of the micromotor. The micromotor was analyzed in both time and frequency domain. The electric potential global variable—configuration variable—has been calculated. For comparison purpose, an analytical solution of the electric potential is utilized as reference. Comparisons against analytical solutions of the electric potential demonstrated the cogency of our proposal. In frequency domain, the error between analytical and CM is less than 0.3‰—for a primal mesh of 2,353 nodes and 4,704 elements. In addition, transient analysis in time domain has been carried out using θ-method. Electric potential at the interface of the micromotor tends to the steady state value validating our approach, also.

## Figures and Tables

**Table 1.** Nomenclature.

**Symbol**	**Name**	**Unity**

*a*	Height of the air gap	m
*b*	Height of insulator	m
*k*	Number of waves per metre	-
*l*	Length	m
*j*	Imaginary unity	-
*J_f_*	Current density	A/m^2^
*S*	Slip	-
*t*	Thickness	m
*ν*	Linear speed of mobile part	m/s
*V*	Interelectrodic potential	V
*V_0_*	Supply potential	V
*ε_a_*	Electric permittivity of the air	F/m
*ε_b_*	Electric permittivity of the insulator	F/m
*ε_eff_*	Effective permittivity	F/m
*φ*	Electric scalar potential	V
*ω*	Angular frequency of the signal	Hz
*σ_a_*	Electric conductivity of the air	S/m
*σ_b_*	Electric conductivity of the insulator	S/m
*σ_S_*	Superficial electric conductivity	1/Ω
*σ_eff_*	Effective Conductivity	S/m
*Φ^b^*	Potential at the interface	V

**Table 2.** Physical and geometrical parameters of the micromachine.

**Symbol**	**Name**	**Value**	**Unit**

*L*	Length of the structure	44	μm
*h_m_*	Height of the metal sheet	0.01	μm
*a*	Height of dielectric 2	3	μm
*b*	Height of dielectric 1	10	μm
*k*	Number of waves per meter	2π/L	μm^−1^
*ν*	Linear speed of mobile part	0	μm/s
*f*	Temporal frequency of excitation	2.6 × 10^6^	Hz
*V_0_*	Maximum value of excitation	200	V

**Table 3.** Interface electrical potential.

**Conductivity 1/Ω**	**Analytical solution V**	**CM V**	**Error %**

1/(50·10^6^)	21.6688	21.6947	−0.119
1/(100·10^6^)	37.7909	37.7259	0.172
1/(200·10^6^)	53.6311	53.5904	0.075
1/(600·10^6^)	64.2738	64.2748	−0.001
1/(1800·10^6^)	65.8906	65.9102	−0.029

**Table 4.** Electric field in the steady state at the interface in z = 0.

**Conductivity 1/Ω**	**Analytical solution V/m**	**CM V/m**	**Error %**

1/(50·10^6^)	3094307	3102000	−0.248
1/(100·10^6^)	5381641	5389700	−0.149
1/(200·10^6^)	7658503	7665400	−0.090
1/(600·10^6^)	9178278	9182800	−0.049
1/(1800·10^6^)	9409100	9419900	−0.114

**Table 5.** Effect of the mesh in the convergence.

**Number of nodes**	**Number of elements**	**Analytical solution V**	**CM V**	**Error %**
2353	4704	65.89	65.91	0.030
613	1224	65.89	66.02	0.197
284	566	65.89	66.20	0.470
170	338	65.89	66.40	0.774
